# A Simple and Practical Single-Camera Stereo-Digital Image Correlation Using a Color Camera and X-Cube Prism

**DOI:** 10.3390/s19214726

**Published:** 2019-10-31

**Authors:** Bo Dong, Fancang Zeng, Bing Pan

**Affiliations:** 1 Institute of Solid Mechanics, School of Aeronautic Science and Engineering, Beihang University, Beijing 100191, China; bdong@buaa.edu.cn (B.D.); zengfancang@buaa.edu.cn (F.Z.); 2 Beijing Advanced Discipline Center of Unmanned Aircraft System, Beihang University, Beijing 100191, China

**Keywords:** stereo digital image correlation, color camera, X-cube prism, deformation measurement

## Abstract

A simple and practical full-frame single-camera stereo-digital image correlation (stereo-DIC) technique for three-dimensional (3D) shape, displacement, and deformation measurements is proposed. The technique uses a compact X-cube prism-based color separation device and a color camera to capture images of blue and red colors from different optical paths, and then extracts the surface 3D shape and deformation information of a test sample by processing the captured two sub-channel color images using regular stereo-DIC algorithm. Compared with the existing full-frame single-camera stereo-DICs, the proposed one eliminates the need for a beam splitter and two bandpass filters to capture images, and offers more simple, compact, and easy-to-use optical arrangement. This novel single-camera stereo-DIC technique was validated by a series of baseline experiments involving 3D surface reconstructions, translation tests, and full-field deformation measurements, which provide a new flexible and practical avenue for measuring surface 3D shape and deformation, particularly in microscopic and high-speed applications.

## 1. Introduction

Stereo-digital image correlation (stereo-DIC) is an image-based non-contact optical technique for retrieving full-field 3D shape, displacement and deformation of materials, structures, and biological trusses [1]. In recent years, significant advances have been made to various aspects of stereo-DIC to improve its robustness, applicability, measurement accuracy, and computational efficiency. As a matter of fact, stereo-DIC has been widely accepted as the most powerful tool in the experimental mechanics community, and it offers several prominent advantages over competing interferometric optical techniques (e.g., electronic speckle pattern interferometry and Moiré interferometry [2]), such as being easy-to-use, a robustness against ambient vibrations, and a wide range of applicability.

The conventional stereo-DIC technique uses two synchronized digital cameras to acquire digital images of a test object from different views and then retrieves the 3D information from the stereo pairs using image registration algorithm and triangulation [1]. However, the requirement of two synchronized cameras usually makes the system both expensive and complicated, especially for the high-speed stereo-DIC measurements. To improve the stereo-DIC, various single-camera stereo-DIC techniques using diffraction-based, refraction-based, and reflection-based optical configurations have been developed and advocated [3]. Among these techniques, full-frame single-camera stereo-DIC technique using a color separation device and a color camera has been considered the most practical one, since it can realize full-frame measurements without any loss of spatial resolution of the camera, while maintaining the prominent advantages of other single-camera stereo-DIC techniques such as cost-effectiveness, compactness, and the avoidance of the complicated camera synchronization [4,5]. 

**Figure 1:**
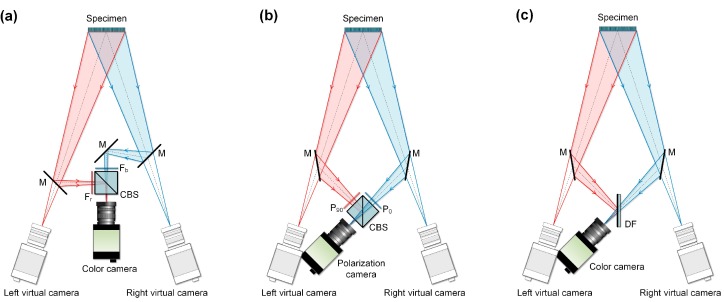
Optical arrangements of (**a**) color separation device-assisted single-camera stereo-digital image correlation (stereo-DIC), (**b**) polarization-based single-camera stereo-DIC, (**c**) dichroic filter-aided single-camera stereo-DIC. M: Mirror, CBS: cube beam splitter, F_b_: blue bandpass filter, F_r_: red bandpass filter, P_90_: 90° polarizer, P_0_: 0° polarizer, DF: dichroic filter.

The full-frame single-camera stereo-DIC technique using a single-color camera was originally proposed by Yu and Pan [4]. Specifically, they designed an innovative color separation device to facilitate a color camera to acquire images of blue and red colors from different optical paths, and then the 3D information could be retrieved from the extracted two-channel sub-images. Figure 1a gives the optical setup of the method, where a cube beam splitter (CBS) is used to combine two different views reflected from two plane mirrors (M) and the color filters (F_b_ and F_r_) arranged in front of the CBS is used to remove the color cross-talk between the two views. Benefitting from its full-frame 3D measurement capability, the technique was then successfully employed for high-speed displacement and deformation measurements in rotation, vibration, and explosion tests [6]. Very recently, this technique was further extended to microscopic scales for characterizing mechanical behaviors of small-size specimens with micro features [7]. Inspired by the novel idea proposed in [4], several alternative full-frame single-camera stereo-DIC systems were also established, which use other forms of light separation devices instead of using a CBS and two-color filters. For instance, a polarization camera and two polarizers were used to achieve the same function (i.e., full-frame stereo-DIC measurement using a single camera) [8]. As shown in Figure 1b, the optical setup of the method is similar to the color separation device assisted one, but it uses two polarizers with different polarization angles to remove the interference between the two views. Recently, a seemingly simpler dichroic filter (DF) [9] was also used as a color separation device to realize full-frame single-camera stereo-DIC measurement as shown in Figure 1c.

Despite the fact that the feasibility and functionality of these existing single-camera stereo-DIC systems have been well demonstrated in previous works, they do have several following shortcomings. Regarding the first optical configuration shown in Figure 1a, it involves too many optical elements, which makes the optical system both cumbersome and not easy to use. In respect to the polarization-based color separation device shown in Figure 1b, the practicality and applicability of the technique are very limited because of the high cost of, and inaccessibility to, high-speed polarization cameras [8]. As for the dichroic filter-assisted color separation device, this simplification brings serious imaging problems. First, the ghost image reflected from the rear surface of the DF will degrade the imaging quality of the blue channel. Second, the refraction effect presented when the blue light passes through the DF can lead to considerable measurement error [9]. 

To overcome the limitations of the existing single-camera stereo-DIC systems, a simple and practical solution based on the use of X-cube prism is proposed in this work, which greatly improves the compactness and operability of the full-frame single-camera stereo-DIC system. First, it overcomes the imaging problems existed in the DF-assisted system while maintains its extreme simple optical setup. Second, the symmetrical structure of the X-cube prism-based color separation device also makes the system very compact and easy to adjust. Because both the performance and operability of this newly developed single-camera stereo-DIC are very close to that of the conventional binocular one, this technique can be considered as the best single-camera solution for stereo-DIC measurements.

In the remainder of this manuscript, the system configuration and implementation procedure of the technique are first introduced. After that, X-cube prism assisted single-camera stereo-DIC systems are established and the validation experiments are detailed. Finally, the contours of curved surfaces with complex structures and the deformation fields of specimens under different loadings are successfully measured to exhibit the practicability of the method.

## 2. Measuring System and Principles

### 2.1. System Configuration

**Figure 2:**
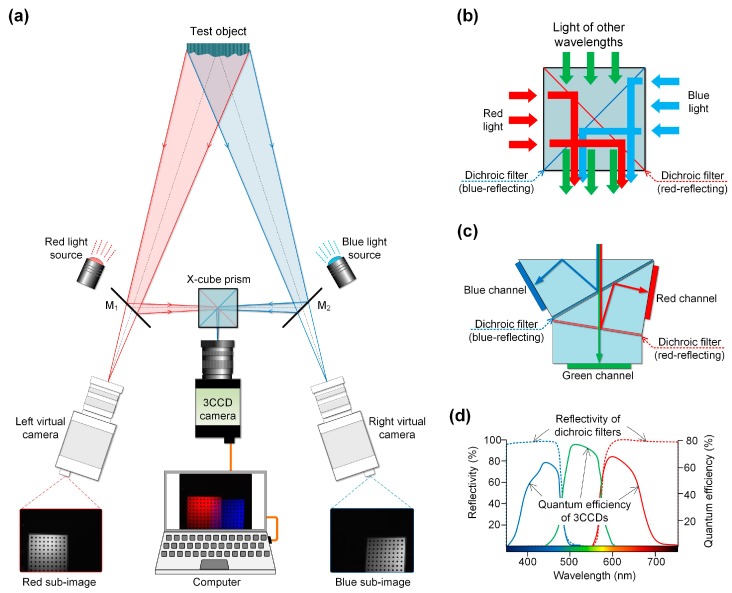
Schematics of (**a**) the proposed X-cube prism assisted single-camera stereo-DIC system, (**b**) an X-cube prism, (**c**) a 3CCD color camera, (**d**) reflectivity of the dichroic filters and quantum efficiency of 3CCDs.

The schematic of the X-cube prism assisted single-camera stereo-DIC system is shown in Figure 2a. It consists of an X-cube prism, two symmetrically placed plane mirrors (denoted as M_1_ and M_2_), a color camera connected with an imaging lens, and a computer. The X-cube prism, which is composed of two DFs (a blue- and a red-reflecting one) as shown in Figure 2b, is generally employed in digital light processing (DLP) projector system for recombining red, green, and blue color components of the light [10]. In this system, it is used to collect red light comes from the left side and blue light comes from the right side. Due to the symmetrical structure of X-cube prism, the compactness and operability of the single-camera stereo-DIC system can be significantly improved, and the ghost image and refraction effect intrinsic to a dichroic filter can be effectively avoided. It should be noted that to capture color images, a Bayer filter-based color complementary metal oxide semiconductor (CMOS)/ charge-coupled device (CCD) camera or a three-CCD devices (3CCD) color camera should be used in combination with the color separation device.

However, in addition to some special requirements, e.g., high-speed measurement requirements, a 3CCD color camera is highly recommended due to its better capability for avoiding the cross-talk between different color channels [11,12]. The schematic of the 3CCD color camera is shown in Figure 2c, it is composed of three separate charge-coupled devices with each one taking a separate measurement of the primary colors. Figure 2d gives the reflectivity of the dichroic filters of the X-cube prism and the quantum efficiency of the 3CCD color camera, from which it is seen that the cross-talk between the captured images of different views can be effectively avoided by combining the X-cube prism and 3CCD color camera. In practical use, the system can also be equipped by a tunable blue- and red-light source, so that the average grayscale of the collected blue and red sub-channel images can be kept at the same level during measurements.

Compared with the existing full-frame single-camera stereo-DIC systems, the major advantages of this newly developed one can be concluded as the following two aspects: (1) It uses an X-cube prism to replace the previous CBS, F_b_, and F_r_, which makes its optical arrangement much simpler and more compact than that of the previous systems without introducing any imaging problems; (2) the baseline, working distance, and virtual pan angle of the system can be easily altered by translating and rotating the two plane mirrors just like adjusting the two cameras of a binocular stereo-DIC system. In a word, this newly developed single-camera stereo-DIC system has better compactness and operability than that of the previous versions, which can replace single-camera and binocular stereo-DIC systems in most measurement scenarios.

### 2.2. Working Principle and Implementation Procedure

**Figure 3:**
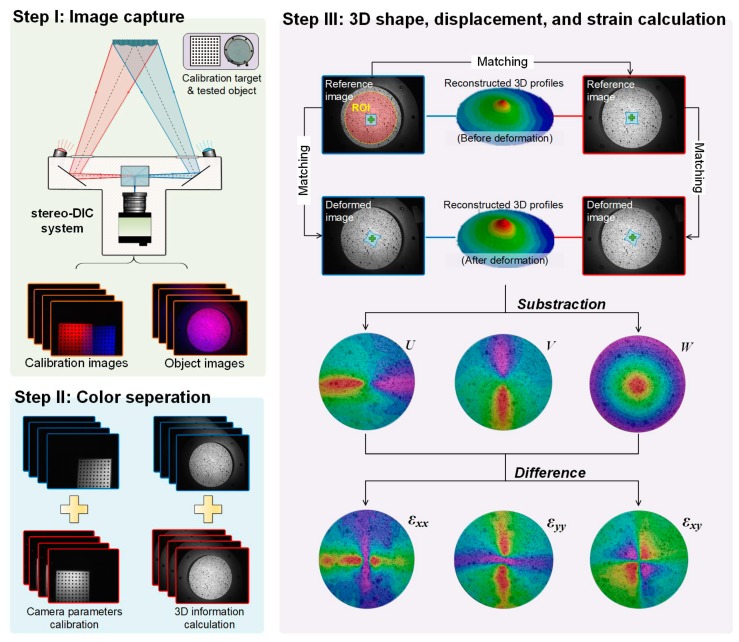
Schematic of the implementation procedure.

The working principle and implementation procedure of this technique, as shown in Figure 3, is the same as our previously proposed color separation device assisted full-frame single-camera stereo-DIC [4]. During measurement, the color images of the test object and a calibration target are first captured by the X-cube prism assisted single-camera stereo-DIC system. Then, the captured color images are separated into red and blue sub-images to calibrate the camera parameters and calculate the 3D shape, displacement, and strain information. It should be noted that, if a Bayer filter-based color CCD/CMOS camera is employed by the measuring system to capture color images, mainly in the high-speed measurement situations, the cross-talk between the different color channels will have to be addressed [6]. In the calculation procedure, as shown in “Step III” of Figure 3, a subset centered at each calculation point is first selected from the blue channel sub-image of the initial state to search its target in the corresponding red channel one. Then, the desired disparity data of the initial state can be estimated to reconstruct the profile of the region of interest (ROI) at initial state. During the subset search process, a zero-mean normalized sum of squared difference criterion (ZNSSD) [13] is employed to quantitatively evaluate the similarity between the reference and target subsets, and an inverse compositional Gauss-Newton (IC-GN) algorithm [14,15] is used for optimizing the nonlinear ZNSSD criterion. After retrieving the deformed profile within the ROI, the displacement and strain fields can be calculated. Because this calculation procedure is based on the regular stereo-DIC algorithm, it can also be conveniently accomplished by using commercial DIC software.

## 3. Experimental Validation

To validate the effectiveness and accuracy of the proposed method, both regular and microscopic stereo-DIC systems were established. During validation experiments, the profiles of a regular ball surface, a human face model, and a 1-jiao coin were firstly measured to verify the accuracy and practicability of the method for 3D shape reconstructions; then, in-plane and out-of-plane translation tests were carried out to validate the accuracy of the method in displacement measurements; finally, the deformation and strain fields of a rubber membrane and a small round bar under different loadings were measured to show the effectiveness of the method in investigating mechanical behaviors and characterizing mechanical properties.

### 3.1. Measuring Systems

**Figure 4:**
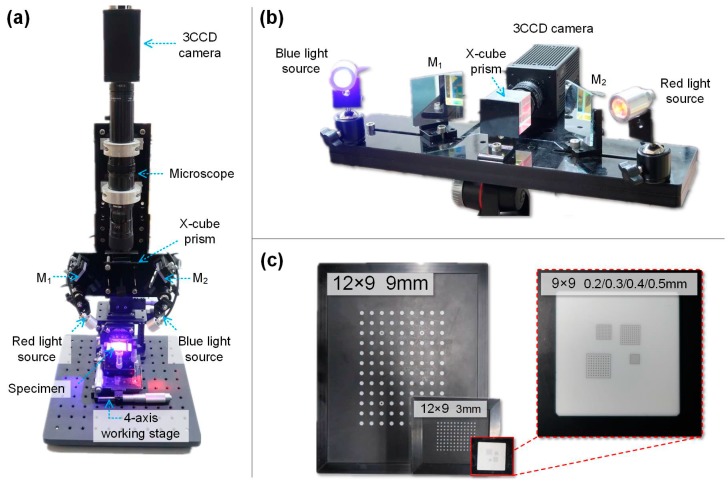
Photographs of (**a**) the established microscopic stereo-DIC system, (**b**) the established regular stereo-DIC system, and (**c**) calibration plates.

The established X-cube prism assisted full-frame single-camera stereo-DIC systems are shown in Figure 4a,b. The systems consist of a 3CCD color camera (AT-200GE, JAI Ltd., Kanagawa, Japan; sensor: 3 × 1/1.8”, active pixels: 1624 × 1236), a high magnification zoom lens (12X Zoom, Navitar Inc., NY, USA; lens attachment: 0.25 ×, adapter: 2 ×, magnification: 0.29–3.5×; working distance: 341 mm) or a regular imaging lens (focal length of 25 mm), a blue- and a red-light source (center wavelengths of 465 nm, and 625, respectively; bandwidth of 10 nm), an X-cube prism (Nanyang Jingliang Photoelectric Ltd., Nanyang, China; size of 30 mm × 30 mm × 30 mm), and two plane mirrors. Figure 4c shows photographs of the calibration targets for the measuring systems. Specifically, an alumina ceramic target containing four small size calibration targets were specially fabricated to calibrate the microscopic measuring system [7]. To calibrate the system parameters and calculate the 3D shape displacement and deformation, commercial software (PMLAB DIC-3D, Nanjing PMLAB Sensor Tech Co., LTD, Nanjing, China) was employed.

It is worth noting that, there are two main reasons that we chose to use blue- and red-light sources rather than a white-light one in our system. First, the red- and blue-light components of a white-light source are generally not the same. Therefore, the gray-scale of the captured blue and red channel images are always different, which will lead to the reduction of accuracy and resolution of the measurement. Second, because there are many other components that exist in a white-light source in addition to the red- and blue-light ones, the illumination efficiency of a white-light source is generally very low for a color-separation device assisted single-camera stereo-DIC system. Therefore, the exposure time of the system must be adjusted to a relatively high level, which will further lead to a low sampling rate or imaging blur.

### 3.2. Morphology Measurement

**Figure 5:**
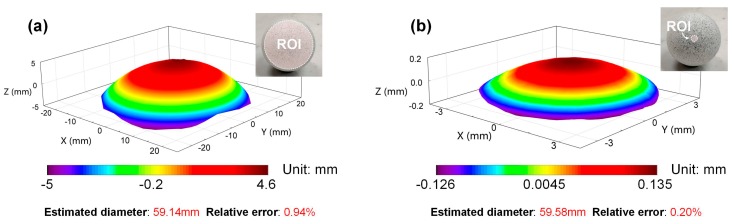
Reconstructed 3D shapes of the ball surface (**a**) using the regular stereo-DIC system, (**b**) using the microscopic stereo-DIC system.

An acrylic ball with a diameter of 59.70 mm was first measured using the established stereo-DIC systems to verify the precision of the proposed system in 3D shape reconstruction. Before the measurement, random speckle patterns were first decorated onto the ball surface by spraying white and black paints using spray bottles. The dot sizes of the speckle patterns are ranging from 0.01 to 1 mm. Then, the illumination intensity of the light source and the exposure time of the 3CCD camera were adjusted to capture clear images of the ball surface. After separating the color images captured by the measuring system, a circular region of interest (ROI) was firstly specified in the blue channel sub-image. Matching the blue and red channel sub-images using the DIC algorithm, the profile of the ball surface can be successfully reconstructed. The reconstructed profiles of the ball surface shown in Figure 5a,b correspond to the results measured using the regular and microscopic measuring systems, respectively. By fitting the reconstructed 3D point clouds with fast geometric fit algorithm, the diameters of the ball surface can be estimated. Compared with the reference value, the relative errors of these two measurement results can be estimated, which are 0.94% and 0.20%. The relative errors are close to that of the existing full-frame single-camera stereo-DIC techniques (relative errors less than 1%) [4,7], indicating the established single-camera stereo-DIC method can be employed for accurately measuring curved 3D surfaces.

**Figure 6:**
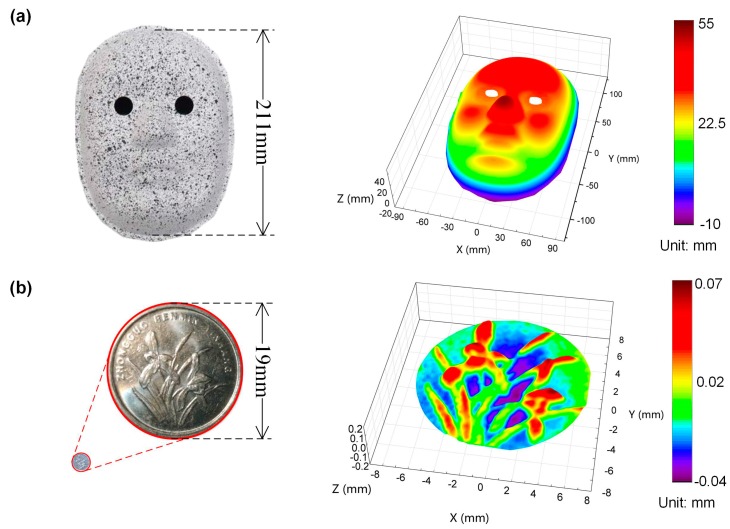
Photographs and reconstructed profiles of (**a**) a human face model and (**b**) a 1-jiao coin.

To further investigate the practicability of the proposed stereo-DIC system in 3D shape reconstructions, the profile of a human face model, as shown in Figure 6a, was measured. From the measurement result, it is seen that the 3D structures of forehead, nose, cheeks, lips, and jaw of the face model have been perfectly reconstructed. Then, the rear surface of a 1-jiao coin was also measured as shown in Figure 6b, from which it is seen that even sub-millimeter scale details of the 3D orchid pattern have been successfully reconstructed. The results of these two experiments indicate that the novel technique can be conveniently and effectively employed for 3D shape reconstructions in different needs of practical tests.

### 3.3. In-Plane and Out-Of-Plane Translation Test

**Figure 7:**
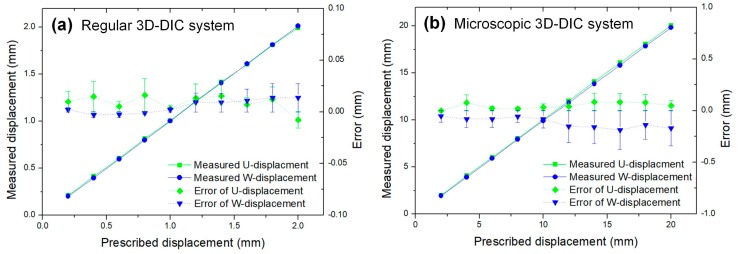
Experimental results of in-plane and out-of-plane translation tests (**a**) regular stereo-DIC measuring system and (**b**) microscopic 3D-DIC measuring system.

To verify the displacement measurement accuracy of the proposed stereo-DIC systems, in-plane and out-of-plane translation tests were carried out. First, a two-dimensional linear translation stage, with a flat plate fixed on it, was placed in front of the measuring system. Then, we controlled the rigid-body movement of the plate using the translation stage and measured the displacement of the plate using the established measuring system. After that, the displacement measurement accuracy of the proposed method can be validated by comparing the prescribed and measured displacements. During the DIC calculations, a rectangular region located at the plate within the blue channel sub-image, containing 2601 (51 rows × 51 columns) discrete points, was chosen to be the ROI. To better verify the displacement measurement accuracy, the translation tests were carried for each measuring system. Specific to the regular stereo-DIC system, the movement of the plate in both the in-plane and out-of-plane has been tested from 0 to 20 mm with 2 mm intervals. The measurement result of the experiment is shown in Figure 7a, from which it is seen that the measured displacements are in perfect agreement with the prescribed ones, and the absolute value of the mean error is less than 0.1 mm. For the microscopic stereo-DIC system, the movement of the plate in both the in-plane and out-of-plane has been tested from 0 to 2 mm with 2 mm intervals. Figure 7b gives the prescribed and measured displacements, from which it is observed that the absolute value of the mean error is less than 0.01 mm. From both of these two experiments, it is noticed that the displacement error is close to that of the existing full-frame single-camera stereo-DIC techniques [4,7] (relative errors less than 0.5%), which indicates that the technique can perform high-accuracy displacement measurement.

### 3.4. Deformation Field and Strain Field Measurement

**Figure 8:**
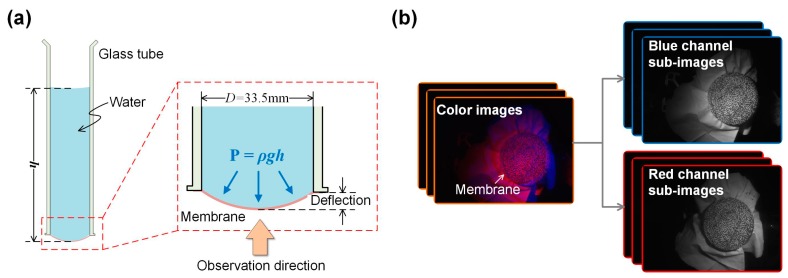
(**a**) Schematic of the nitrile butadiene rubber (NBR) testing device and (**b**) captured color images and separated blue and red channel sub-images.

To investigate the effectiveness of the proposed method in characterizing mechanical behaviors, the deformation and strain fields of a nitrile butadiene rubber (NBR) membrane (thickness *t* = 0.10 mm) under uniform press loading were measured using the established regular stereo-DIC system. As shown in Figure 8a, the tested NBR membrane was fixed to the bottom of a glass tube, and therefore the pressure P applied onto the membrane can be easily controlled by adjusting the height *h* of the water level. After carefully adjusting the light source and measuring system, the color images of the membrane were captured when *h* reaches 10, 20, …, 100 mm, respectively. During the DIC calculations, a circular region with a diameter of ~30 mm, was chosen to be the ROI, a subset size of 51 × 51 pixels and a grid step of 5 pixels were chosen as the calculation parameters for each point of interest to estimate the displacement and strain fields of the deformed membrane. Considering the water density and gravitational acceleration are *ρ* = 10^3^ kg/m^3^ and g = 9.8 m/s^2^, the pressure value of the initial state (preloading value) is of 0.098kPa.

**Figure 9:**
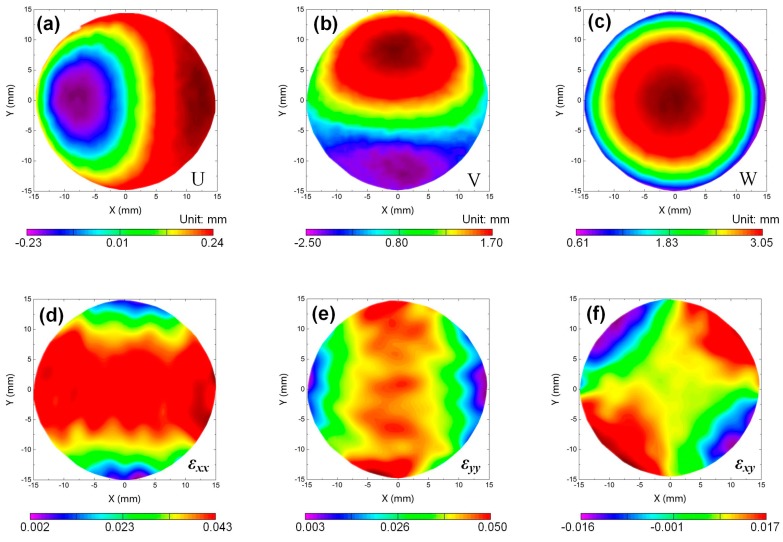
Experimental results of the centrally loaded circular plate (**a**) U-displacement field, (**b**) V-displacement field, (**c**) W-displacement field, (**d**) *ε_xx_*-strain field, (**e**) *ε_yy_*-strain field, and (**f**) *ε_xy_*-strain field.

The calculated full-field U, V, and W components of the surface deformation of the membrane when *h* = 100 mm are shown in Figure 9a–c, respectively. From these results, it is seen that the U/V-displacement field exhibits an antisymmetric distribution with respect to *y*/*x* axis, and it features zero displacements along the *x*/*y* axis. The W-displacement field presents a concentric pattern with the center located at the central point of the membrane. The maximum displacement in the W-displacement field is 3.05 mm. Figure 9e–f shows the in-plane strain components *ε_xx_*, *ε_yy_,* and *ε_xy_* estimated from the measured displacement fields, from which symmetric distributions can also be observed. Since these measured displacements and strain fields can be well explained by the boundary conditions, the effectiveness of this method in displacement and strain field measurement can be confirmed.

**Figure 10:**
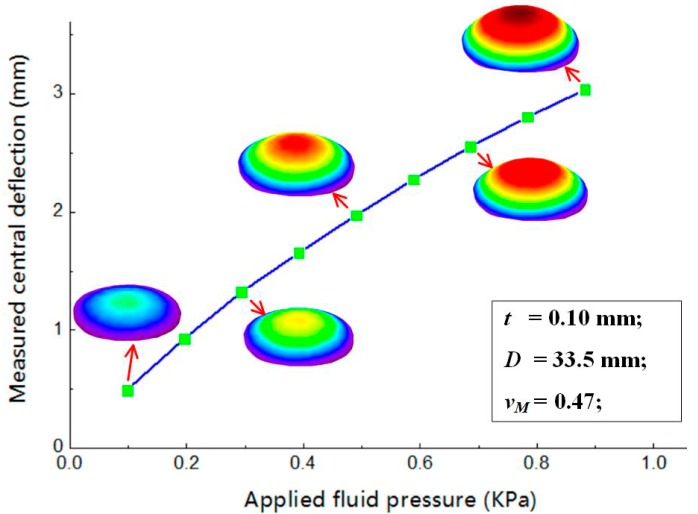
The relation between the measured central deflection *w*_0_ and applied fluid pressure P.

To further estimate Young’s module *E_M_* of the measured NBR membrane, the relationship between the central deflection *w*_0_ and applied fluid pressure P, as shown in Figure 10, was then acquired based on the measured W-displacement fields. Theoretically, the relationship between them can be regarded as P = *aw*_0_^3^ + *b*w_0_. Therefore, by fitting the measured values using the function, the coefficients can be estimated, which are *a* = 8.25×10^9^ and *b* = 1.54×10^5^. After that, Young’s module of the membrane can be determined by the formula [16]

(1) $$E_{M}=\frac{3a\cdot D^{4}(1.026-0.793v_{M}-0.233v_{M})}{8t},$$

where *v_M_* is the Poison’s ratio of the membrane. By substituting the parameters into the equation, Young’s module of this membrane can be calculated as 2.035 MPa, which is consistent with its reference value (2.188 MPa, measured by a tensile test). From this experiment, it is validated that this method can be effectively employed for investigating mechanical behaviors and characterizing mechanical properties of materials and structures.

**Figure 11:**
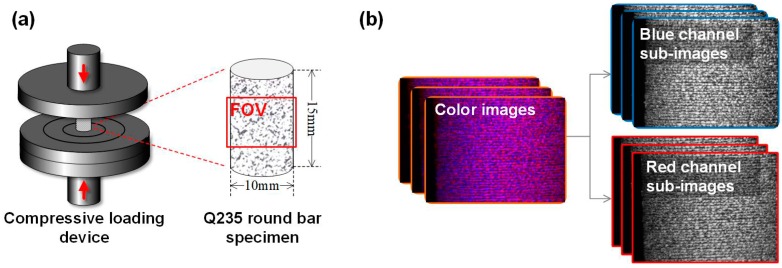
(**a**) schematic of the compressive loading device and (**b**) captured images and separated blue and red channel sub-images.

Finally, to validate whether the proposed method can be effectively applied in visualizing mechanical behaviors and characterizing mechanical parameters of small specimens, a uniaxial compression test was conducted using the established single-camera microscopic stereo-DIC system. Before the experiment, a Q235 round bar specimen (diameter and height are of 10 and 15 mm, respectively) was clamped with 1 KN preload on a compressive loading device as shown in Figure 11a. Then, adjusting the light source and the measuring system to capture clear images of the specimen during its compression procedure. During the experiment, the upper platen of the compressive loading device was controlled to move down with a speed of 0.02 mm/s. The captured color images and separated blue and red channel sub-images are shown in Figure 11b.

**Figure 12:**
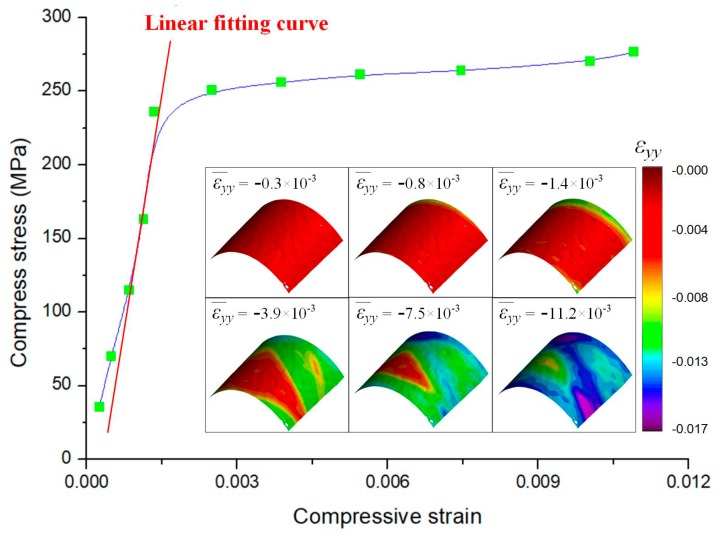
Stress–strain curve of the Q235 round bar specimen.

According to the force sensor reading, the cross-sectional area of the specimen, and the strain distribution measured using the system, the stress-strain curve of the specimen can be plotted as shown in Figure 12. The strain field distributions *ε_yy_* corresponding to different loading states can be found in the right bottom corner of the figure. From the curve, it is seen that the yield strength of the specimen is about 235 MPa, which consistent with its reference value (235 MPa) [17]. By linear fitting, the measured results before specimen yield, Young’s modulus of the specimen can be estimated as 204.4 GPa, which is within the range of the reference value (196–216 GPa). This experiment indicates that the proposed method can be effectively applied to the microscopic measurements to realize accurate mechanical characterization.

## 4. Conclusions

In this work, a simple and practical full-frame single-camera stereo-DIC technique is proposed for measuring 3D shape, displacement, and deformation. It uses a compact X-cube prism-based color separation device and a color camera to capture images of blue and red colors from different optical paths, and then extracts the surface 3D shape and deformation information using the regular stereo-DIC algorithm. The accuracy, effectiveness, and practicality of the established single-camera stereo-DIC have been verified by a series of validation experiments. This novel technique not only maintains all the advantages associated with existing full-frame single-camera stereo-DIC, such as low cost, no need for camera synchronization, and full-frame measurement capability, but offers special advantages of much simpler, more compact, and easy-to-adjust optical configuration as well. It demonstrates great potential in investigating mechanical behaviors of materials and structures in different scenarios, particularly in high-speed and microscopic applications.
